# Effect of Group Mindfulness-Based Stress-Reduction Program and Conscious Yoga on Lifestyle, Coping Strategies, and Systolic and Diastolic Blood Pressures in Patients with Hypertension

**Published:** 2015-07-03

**Authors:** Somayeh Nejati, Alireza Zahiroddin, Gita Afrookhteh, Soheila Rahmani, Shahrzad Hoveida

**Affiliations:** 1 *Behavioral Sciences Research Center, Shahid Beheshti University of Medical Sciences, Tehran, Iran.*; 2 *Azad University of Sciences and Research, Tehran, Iran.*; 3 *Department of Psychology and Educational Sciences, Semnan University, Semnan, Iran.*; 4 *Alborz Azad University of Sciences and Research, Karaj, Iran.*

**Keywords:** *Intervention studies*, *Yoga*, *Mindfulness*, *Hypertension*, *Life style*

## Abstract

**Background:** Healthy lifestyle and ineffective coping strategies are deemed significant variables among patients with hypertension. This study attempted to determine the status of these variables following intervention via the mindfulness-based stress-reduction program (MBSRP) in patients with hypertension.

**Method**: This study was a randomized clinical trial. The study sample, consisting of 30 patients referring to the Hypertension Clinic of Imam Hossein Hospital in 2013, was assigned either to the intervention (recipient of the MBSRP and conscious yoga) or to the control group (recipient of yoga training). The intervention group had 8 training sessions over 8 weeks. Lifestyle and coping strategies as well as blood pressure were measured in the intervention group before intervention and then immediately thereafter and at 2 months' follow-up and were compared to those in the control group at the same time points.

**Result**: The mean age of the patients in the intervention (40% women) and control (53% women) groups was 43.66 ± 5.14 and 43.13 ± 5.04 years, respectively. The results showed that the mean scores of lifestyle (p value < 0.05), emotion-focused coping strategies (p value < 0.001), problem-focused coping strategies (p value < 0.001), diastolic blood pressure (p value < 0.001), and systolic blood pressure (p value < 0.001) were significantly different between the intervention and control groups after the intervention.

**Conclusion: **Applying an intervention based on the MBSRP may further improve the lifestyle and coping strategies of patients with hypertension.

## Introduction

Hypertension is the third cause of death in the world.^1^ The results of studies conducted in Iran and elsewhere show that the prevalence of hypertension around the globe, and in particular in Iran, is on the rise.^2^ The disease is known as the "silent killer" because there are no apparent and recognizable signs and there are unpleasant cardiovascular complications resulting from the disease.^1^^, ^^2^ The aims of the Health Project (2010) consisted of dealing with hypertension, decreasing its incidence, and increasing chances of success in its treatment and control. Achieving these goals requires the application of a multidimensional approach. Although drug therapy is important in blood pressure control, the patient's lifestyle is a factor exerting a significant impact on its control.^3^ Hypertension can lead to increased risk of heart disease, myocardial infarction, stroke, and kidney diseases.^1^^, ^^4^ In most cases, the actual cause of hypertension is unknown, but in general, diseases and lifestyle (i.e. lack of regular activity, inappropriate nutrition, smoking, and alcohol consumption) have a major effect on decreasing the quality of life and increasing the incidence of the disease.^3^^, ^^4^ It seems that lifestyle and stress and its coping strategies are the factors correlated with the incidence and progression of hypertension.^5^

Lifestyle is defined as a way of life that an individual chooses, with its foundation laid in family and affected by the individual's culture, race, and economic and social status.^6^ Recent studies have shown that lifestyle can cause stress, anxiety, and mental pressure and consequently bring about a rise in the blood pressure and other chronic diseases.^5^^, ^^7^ Ariff et al.,^5^ believing that improving lifestyle components can help individuals to stay healthy and cope with daily stresses, posited that a healthy lifestyle can have an effective role in individuals' happiness and prevention of stress and depression. Health training focuses on lifestyle, seeking to change individuals' behaviors and motivating them to develop a healthy lifestyle.^8^ In this regard, Hung et al.^6^ demonstrated that lifestyle modification is considered one of the foremost therapeutic strategies in treating patients with hypertension. Indeed, research has demonstrated that lifestyle change is effective in improving or controlling chronic diseases such as hypertension.^3^

The results of previous studies have revealed that almost all diseases have psychosomatic components and that any kinds of diseases, particularly heart diseases and hypertension, are affected by stress and stressful events.^9^ Accordingly, alleviating stressful factors is an effective technique in reducing the blood pressure. Although the existence of stress is inevitable and having adequate levels of stress confers growth and development, high levels of stress or ineffective coping strategies are harmful. Learning effective coping strategies leads to the maintenance and promotion of health.^10^ There are two coping strategies applied by individuals when facing specific problems and diseases such as hypertension: the problem-focused coping strategy and the emotion-focused coping strategy.^11^ In the emotion-focused coping strategy, the objective is to focus on emotion and to control emotional distress. This strategy is more associated with physical exercise, care, expression of feelings, and seeking social support. In the problem-focused coping strategy, the objective for individuals is to perform a problem-solving activity; and if they believe that the situation is beyond their capabilities, they can apply the emotion-focused coping strategy.^11^ Ariff et al.^5^ reported that patients with hypertension tend to apply inefficient coping strategies and that the application of these strategies brings about excess stress and emotional instability and thus mental and physical damage.

In general, previous research has demonstrated that controlling stress and using effective coping strategies can be useful in treating hypertension.^5^^, ^^11^ In previous studies, the psychological consequences of hypertension such as anxiety and depression were mostly considered, but there was less attention to coping strategies with psychological factors and intervention in the lifestyle of patients with hypertension. In this regard, the mindfulness-based stress-reduction program (MBSRP) is one of the treatment modalities deemed effective in individuals' health training against physical and psychological diseases. The MBSRP is based on the three components of avoiding judgment, enhancing awareness, and focusing on the present moment. Paying attention to the present moment helps individuals to process their cognitive, physiological, and behavioral activities. It happens because through the regular practice of mindfulness-based activity, individuals become aware not only of their daily activities but also of the automatic function of their mind in the past and future. Additionally, via the moment-to-moment awareness of their thoughts, feelings, and physical states, individuals will learn to control them and to release themselves from the automatic mind, which is focused on the traumatic past and future.^12^^, ^^13^ According to this approach, a judgmental mind increases the levels of stress, anxiety, and depression in that it tends to divide experiences into good and bad ones. A judgmental mind, therefore, seeks to struggle with or avoid experiences, both of which will result in stress and anxiety.^14^

The most important component of mindfulness practices is the enhancement of awareness, which means commitment to the presence of mind such that the individual can move from one moment to another moment easily without yielding to the judgmental and non-reactive mind and without trying to get anywhere.^15^

Mindfulness-based interventions with the aim of alleviating psychological symptoms and enhancing the quality of life are taken advantage of in psychological and physical health.^15^ Niazi et al. reported that mindfulness reveals internal and external realities freely and without distortion and shows a great capability in dealing with a wide range of thoughts, emotions, and experiences (both pleasant and unpleasant). They also suggested that mindfulness has a positive relationship with mental and psychological calmness and mental health in chronic diseases.^16^ The MBSRP has shown success in treating physical and mental symptoms such as generalized anxiety, panic disorders, eating disorders, and stress symptoms as well as in improving the quality of life in patients with breast and prostate cancer and fibromyalgia.^13^^, ^^15^^, ^^17^

A meta-analysis by Bohlmeijera et al.^15^ showed that the MBSRP is an effective method to reduce stress and improve the quality of life in individuals with a diagnosis of different diseases. This meta-analysis study included patients with cancer suffering from psychiatric disorders, chronic pain, and cardiovascular diseases such as hypertension. The results of the Gloria et al.^18^ study, conducted to demonstrate the effectiveness of the MBSRP in patients with hypertension, showed that this program is an effective treatment both in reducing stress and anxiety in daily life and in reducing the symptoms of chronic diseases such as high blood pressure. Parswani et al.^19^ demonstrated that the MBSRP can reduce the symptoms of anxiety and depression in patients with coronary heart disease and hypertension. Additionally, the authors reported that after a 3-month follow-up, the treatment effects were still present.

Given the high prevalence of hypertension and its complications and treatment costs, it seems that a simultaneous combination of non-pharmacological therapies (both in treatment and in prevention) and drug treatment could at least reduce these undesirable effects and accelerate improvement. To that end, survey investigations are the first step toward planning appropriate non-pharmacological treatment strategies. We, therefore, sought to assess the effect of group MBSRP and conscious yoga on lifestyle, coping strategies, and systolic and diastolic blood pressures in patients with hypertension.

## Methods

This study was quasi-experimental study with a control group and featured pre-test, post-test, and follow-up. The blood pressure of each patient in the two study groups was measured three times. All the patients who referred to Imam Hossein Hospital in Tehran in 2013 with a diagnosis of hypertension were recruited in this study. Among these patients, 30 individuals who met the inclusion criteria, i.e. not receiving psychological treatment at the time of diagnosis, having blood pressure > 130/80 mmHg, having high school diploma or higher qualifications, age between 30 and 55 years, and having similar drug regimen, were included in the study. Patients with mental illnesses and physical diseases such as diabetes, renal disease, liver disease, and history of myocardial infarction were excluded from the present study. Also excluded were pregnant patients and those missing more than 2 intervention sessions. The study population was randomly assigned into experimental and control groups, each consisting of 15 individuals. The adequacy of the sample size was examined by basic statistical methods and the results of the previous studies^20^ with a confidence level of 95% and error of 5%. A significance level < 5% error rate rejected the null hypothesis. After obtaining written informed consent from all the patients, pre-test evaluations were conducted.

***Tools***

***The Clinical Version of the Structured Clinical Interview for Axis-One Disorders in DSM-IV (SCID-I /CV)***

It is a standardized tool for evaluating major psychiatric disorders based on the DSM-IV definitions and criteria designed for clinical and research goals. The implementation of the SCID-I/CV needs clinical judgment of the interviewer about the answers of the interviewee; therefore, the interviewer should have enough knowledge and experience about psychological pathology. The validity and reliability of this instrument is reported to be acceptable in different studies. For example, it was reported higher than 0.7 in the diagnostic reliability of Kappa raters. According to a study, the validity of this instrument was confirmed by specialists and test-retest reliability of 0.95 was reported with a one-week interval.^21^ This form was translated and adjusted by Sharifi et al.^21^ in Iran, and its validity and reliability was confirmed.

***Demographic Information Questionnaire***

This questionnaire includes items such as age, education, and inclusion and exclusion criteria.

***Walker's Health Promoting Lifestyle Questionnaire (1997)***

This questionnaire has 54 items. Its aim is to measure health promoting behaviors and has 6 dimensions (i.e. nutrition, exercise, health responsibility, stress management, interpersonal support, and self-actualization).^22^ Likert's range is used to answer the items. The scores for each item comprise never = 1, sometimes = 2, often = 3, and always = 4. In a study by Mohammad Zeidi et al.,^23^ the reliability of the questionnaire was confirmed. The reliability of the questionnaire was also calculated using Cronbach's alpha.^23^ Cao WJ et al.^22^ reported the reliability of this questionnaire based on documented exploratory factor analysis and its validity based on Cronbach's alpha. Cronbach's alpha coefficient is 0.86 for health responsibility, 0.85 for physical activity, 0.80 for nutrition, 0.86 for self-actualization, 0.87 for interpersonal support, 0.79 for stress management, and 0.94 for the entire questionnaire. The validity of the questionnaire in this study was confirmed using formal reliability and the comments of 2 health specialists. In the next step, the correlation between the scores of the subscales and the total score was calculated to further guarantee the reliability. The correlation coefficients between the total score of the Health Promotion Lifestyle Questionnaire and responsibility, physical activity, nutrition, self-actualization, interpersonal support, and stress management were calculated at 0.78, 0.70, 0.65, 0.71, 0.73, and 0.84, respectively.

***Lazarus' Coping Strategies Methods (WOCQ)***

This questionnaire has 66 items and is devised based on an inventory of the coping strategies proposed by Folkman and Lazarus. This instrument evaluates a wide range of thoughts and actions that individuals apply when they face internal or external stressful situations. This test consists of 8 subscales, namely direct coping, distancing, self-control, seeking social support reassessment, accepting responsibility, escape avoidance, planned problem solving, and positive reassessment. The 4 subscales of seeking social support, accepting responsibility, planned problem solving, and positive reassessment are problem-focused strategies and the 4 subscales of direct coping, distancing, self-control, escape avoidance, and self-control are emotion-focused coping strategies.^24^ Individuals answer the items based on Likert's 4-choice scale, which indicates the frequency of each of the used strategies. In this case, zero indicates "I have not used them."; 1 signifies "I have used them to some extent."; 2 denotes "I have used them most of the time."; and 3 implies "I have used them too much.". The items of Lazarus's Coping Strategies Questionnaire have formal validity because the described strategies are those that individuals report they use in coping with stressful situations.^24^ The reliability of the questionnaire and the use of internal consistency (Cronbach's alpha) was estimated at 84/0.

***The Research Implementation Process***

This study was conducted in the Internal Diseases Division of Imam Hossein Hospital in Tehran by 2 holders of master's degree in psychology familiar enough with the planned intervention. The participants provided informed consent and received reassurances about the confidentiality of their information. The subjects in the experimental and control groups completed the questionnaires in 3 stages: before intervention (pre-test); after intervention (post-test); and 2 months post intervention (follow-up). Group treatment was implemented for 8 sessions, once a week for 90 minutes, based on the MBSRP. The control group received no treatment. Due to ethical issues, the participants in the control group were given CDs about yoga at the termination of the study. To assess the normality of the distribution of the scores and homogeneity of the variances, the multivariate covariance analysis with repeated measures was employed. For the statistical analyses, the statistical software SPSS (version 19.0) for Windows (SPSS Inc., Chicago, IL) was used. The contents of the MBSRP and conscious yoga treatment sessions are presented in Table 1.^25^

**Table 1:** Summary of the operating instruction sessions of the mindfulness-based stress-reduction*

Sessions	Topic
First	Practicing automatic guidance system / learning how to use the present-moment awareness of bodily sensation, thoughts, and emotions in reducing stress / practicing how to eat raisins mindfully, providing feedback and discussing the practice / 3-minute breathing exercise, receiving assignment for the following week and leaflets on the first session and CDs about meditation
Second	Re-examining the body workout / providing feedback and discussing the body workout / practicing mindful breathing meditation / performing yoga stretching exercises / receiving leaflets on the second session and CDs about meditation
Third	Practicing conscious sitting with focus on breathing awareness (sitting meditation) / doing yoga exercises / performing 3-minute breathing exercise / receiving leaflets on the third session and CDs about yoga exercises
Fourth	Re-examining the body workout / doing exercises related to conscious yoga / 5-minute practicing of “seeing or hearing” / re-learning awareness of the body, thoughts, and breathing / receiving leaflets on the 4^th^ session and CDs about meditation
Fifth	Practicing breathing / re-practicing awareness of the body, thoughts, and breathing / learning the concept of stress and identifying one's reactions to stress / examining the effect of awareness of pleasant and unpleasant events on feelings, thoughts, and bodily sensations / practicing conscious yoga exercises / practicing 3-minute breathing exercise / receiving leaflets
Sixth	Practicing conscious yoga / practicing sitting meditation (mindfulness of sounds and thoughts) / receiving leaflets on the 6^th^ session and CDs on meditation
Seventh	Learning mountain meditation / sleep hygiene / repeating exercises of the previous session / making a list of enjoyable activities / receiving leaflets on the 7^th^ session
Eighth	Examining the body workout / reviewing the program / examining and discussing the programs / learning stone, bead, and marble meditation

* Reprinted from Kabat-Zinn J: Mindfulness-based interventions in context: past, present, and future. Clinical Psychology: Science and Practice, 2003; 2: 144-156, with permission from John Wiley & Sons.^25^

## Results

The demographic characteristics of the study population are listed in Table 2. There was no significant difference between the control and experimental groups in terms of the mean of the demographic characteristics. The participants' age ranged between 38 and 48 years. The mean age of the patients in the intervention and control groups was 43.66 ± 5.14 and 43.13 ± 5.04 years, respectively.

**Table 2:** Patients characteristics*

	Intervention Group (n=15)	Control Group (n=15)
Sex		
Woman	6 (0.40)	8 (0.53)
Man	9 (0.60)	7 (0.46)
Education		
Third grade of high school	3 (0.20)	3 (0.20)
High school diploma	7 (0.46)	6 (0.40)
Bachelor	5 (0.33)	6 (0.40)
Marital status		
Single	0	0
Married	15 (100)	15 (100)

* Data are presented as n (%).

The mean and standard deviation of the lifestyle, coping strategies, and blood pressure scores in the experimental and control groups in the three stages of pre-test, post-test, and follow-up are reported in Table 3. The data indicate high mean scores of lifestyle and coping strategies and low mean scores of blood pressure in the experimental group in the post-test and follow-up compared to the pre-test. The mean scores of these variables in the control group in the pre-test and follow-up showed no significant changes compared to the pre-test. 

First, the assumptions of using the model were studied. The K-S test results on the normal distribution of the data showed that the distributions of the dependent variables were equal to the theoretical distributions: K-S = 0.83, sig = 0.52 for the lifestyle component; K-S = 0.49, sig = 0.97 for the coping strategy component; and K-S = 1.42, sig = 0.09 for the blood pressure component. The results of the MBox test about the equality of covariance matrices demonstrated that the assumption of the homogeneity of the variance-covariance matrix was established and that the observed covariance matrix of the dependent variables was equal in the groups: F = 2.239; p value = 0.144 for the lifestyle component; F = 0.949; p value = 0.458 for the coping strategy component; and F = 1.949; p value = 0.311 for the blood pressure component. Also, by utilizing Levine's test for the equality of error variances, the assumption of the equality of variances was observed and the error variance of the dependent variable was equal in both groups: F = 2.77, sig = 0.10 for the lifestyle component; F = 1.41, sig = 0.22 for the coping strategy component; and F = 2.80, sig = 0.08 for the blood pressure component. Therefore, the assumptions of the multivariate covariance analysis were used.

**Table 3:** Clinical characteristics of the study population in the two groups of hypertension and non-hypertension*

Indices	Intervention Group (n=15)			Control Group (n=15)		
	Pre-test	Post-test	Follow-up*	Pre-test	Post-test	Follow-up**
Lifestyle						
Nutrition	23.22±1.71	32.00±1.50	31.55±1.42	23.09±4.20	24.46±5.87	24.14±6.10
Exercise	24.05±2.87	31.66±3.24	31.77±4.55	24.04±4.03	24.57±6.36	25.57±6.43
Health responsibility	15.00±3.82	21.77±4.38	20.88±3.51	15.40±4.20	16.42±5.71	15.52±5.22
Stress management	9.44±1.30	14.77±1.64	13.66±1.22	9.23±1.22	9.90±3.57	9.57±3.50
Interpersonal support	11.66±2.06	20.66±1.22	19.77±0.97	11.35±1.87	12.04±5.84	12.04±5.71
Self-actualization	16.55±1.22	23.11±1.45	22.44±1.13	16.61±2.24	16.90±4.17	16.52±4.05
Lifestyle	104.14±26.65	103.38±28.62	140.11±5.79	103.55±26.21	104.33±28.85	144.00±5.63
Coping strategies						
Emotion –focused coping strategies	41.76±8.19	35.46±4.79	35.66±5.10	41.11±7.75	40.42±5.55	41.47±5.12
Problem-focused coping strategies	31.55±5.54	39.93±3.15	38.60±3.08	31.38±4.35	31.46±3.04	30.40±5.21
Blood pressure						
Systolic blood pressure (mmHg)	154.67±7.54	138.11±5.62	135.67±5.80	154.82±5.80	157.16±7.58	155.21±7.21
Diastolic blood pressure (mmHg)	90.58±5.25	86.14±5.65	86.50±5.05	90.34±5.23	92.31±4.51	92.60±5.21

* Data are presented as mean±SD.

** Two months after intervention

**Table 4:** The summary of repeated measures covariance analysis to examine the effect of mindfulness-based stress-reduction on dependent variables

	Sum of Squares	df	Square Means	F	Sig	Eta
Nutrition						
Post-test	54.07	1	54.07	2.398	0.025	0.725
Follow-up	55.763	1	55.763	2.335	0.033	0.718
Exercise						
Post-test	57.303	1	57.30	2.370	0.030	0.718
Follow-up	67.167	1	67.17	2.260	0.035	0.705
Health responsibility						
Post-test	43.66	1	43.66	2.010	0.048	0.678
Follow-up	42.89	1	42.89	2.180	0.045	0.672
Stress management						
Post-test	18.33	1	18.33	2.651	0.037	0.692
Follow-up	17.56	1	17.56	2.443	0.043	0.678
Interpersonal support						
Post-test	52.10	1	52.10	2.392	0.044	0.677
Follow-up	50.36	1	50.36	2.275	0.050	0.589
Self-actualization						
Post-test	33.88	1	33.88	3.241	0.014	0.740
Follow-up	30.38	1	30.38	2.940	0.022	0.728
Lifestyle						
Post-test	1245.69	1	1245.69	3.677	0.017	0.718
Follow-up	1158.70	1	1158.70	3.336	0.023	0.705
Emotion-focused coping strategies						
Post-test	121.48	1	121.48	6.486	0.001	0.575
Follow-up	79.36	1	79.36	3.848	0.001	0.445
Problem-focused coping strategies						
Post-test	117.99	1	117.99	13.091	< 0.001	0.732
Follow-up	105.26	1	105.26	11.738	< 0.001	0.710
Systolic blood pressure						
Post-test	3296.50	1	3296.50	117.849	< 0.001	0.787
Follow-up	31657.48	1	31657.48	116.441	< 0.001	0.754
Diastolic blood pressure						
Post-test	2000.24	1	2000.24	109.405	< 0.001	0.777
Follow-up	1993.42	1	1993.42	107.964	< 0.001	0.742

The results of Table 4 show that the differences between the experimental and control groups in terms of the dependent variables were significant. Thus, in the post-test and follow-up of the experimental group (2 months later), there were significant differences in lifestyle (nutrition, exercise, health responsibility, stress management, interpersonal support, and self-actualization), coping strategies (problem-focused and emotion-focused), and blood pressure (systolic and diastolic) components (p value < 0.05). Consequently, it can be concluded that the MBSRP and conscious yoga were effective on the lifestyle, coping strategies, and systolic and diastolic blood pressures of our hypertensive patients.

## Discussion

Based on our statistical findings, it can be concluded that the MBSRP and conscious yoga in the experimental group, compared to the control group, in both post-test and follow-up had an effect on improving lifestyle in patients with hypertension. The results of the present study indicated that healthy lifestyle behavior training through the MBSRP and conscious yoga decreased blood pressure in the patients and this change was statistically significant (Table 4). 

The findings of the present study are consistent with the results of previously published studies.^26^^-^^29^

The results of a study by de Moraes et al.^27^ showed that lifestyle modifications, including choosing a healthy diet, increasing physical activity, and reducing sedentary behaviors can be beneficial in the management of blood pressure. Additionally, physical activity may contribute to improvements in medication adherence in hypertensives. However, evidence for the effect of lifestyle modifications on lifestyle change and medication persistence is scarce, of poor quality, and suggests little clinically relevant benefit. The results of a study by Lochner et al.^30^ in Portland showed that pharmacotherapy combined with applied training in case of lifestyle change can lead to a reduction of 3 to 11 mmHg in the systolic blood pressure and reduction of 2.5 to 5.5 mmHg in the diastolic blood pressure. 

The results of the present study, however, do not chime in with those of some other studies.^31^ The results of a study by Pandit et al.^31^ in Chicago revealed that health training can increase the knowledge of patients with hypertension, but it has no significant effect on reducing their blood pressure. In contrast, our results showed that lifestyle training through the MBSRP and conscious yoga can reduce psychological symptoms (depression, anxiety, and stress), thereby reducing the patients' blood pressure. A study by Chen et al.^29^ demonstrated the effectiveness of lifestyle training, including sleep and rest, physical activity, and nutrition, in reducing depression, anxiety, stress, and diabetes. Additionally, the meta-analysis found that lifestyle intervention showed significant benefits in risk factors that are known to be associated with the development of cardiovascular disease in patients with type II diabetes. 

Our findings demonstrated that teaching behaviors related to a healthy lifestyle in components such as stress management, interpersonal support, nutrition, and exercise based on the MBSRP and conscious yoga techniques (i.e. concentration, emotion regulation, breathing, and conscious eating) can reduce psychological symptoms (depression, anxiety, etc.) and thus lead to a reduction in the blood pressure in patients with hypertension. The mean of the problem-focused coping strategy in the experimental group had a significant difference with that in the control group in the post-test and follow-up. It can, therefore, be concluded that a combination of the MBSRP and conscious yoga is more effective in patients with hypertension, compared to the control group, in encouraging the use of the problem-focused coping strategy. These findings are consistent with those reported by some previous studies.^32^^-^^34^ Najafi Ghezeljeh et al.^32^ reported that patients with hypertension use emotion-focused coping strategies such as escaping, avoiding stress sources, and refraining from expressing emotions when they face stress. The emotion-focused coping strategy is believed to be effective as a short-term coping strategy; still, in the long term, it endangers mental and physical health. In other words, the use of these strategies by patients with hypertension reduces their ability to solve the problem. Thus, this situation disrupts thought integration, brings about distress, and compromises physical health. In contrast, using problem-focused coping strategies like positive reassessment and emotional and social support strengthens patients against stress and helps them to experience low emotional stress levels. This mental calmness focuses all the abilities of patients on using cognitive skills and dynamics to cope with the problem better. Kabat-Zinn^25^ and Lind et al.^33^ showed that inefficient coping strategies have a negative correlation with mindfulness and that training and increasing mindfulness can decrease inefficient coping strategies. A significant reduction was observed in the post-test in our study population. This can be explained by the notion that judgment causes stress in hypertensive patients in many situations and as such increases the use of the emotion-focused coping strategy and the experiencing of negative emotions and stress. This vicious cycle can lead to an increase in diseases and disorders. To reduce the use of these strategies, it is necessary that patients try not to judge and understand the feelings and events as they are. Generally, treatment positively influenced the patients' illness perceptions, stress-experiences, body- and self-awareness, coping strategies, self-image, social identity, and social functioning. However, the patients identified potentials for treatment improvements, and they needed further treatment to fully recover.

Another result of the present study indicated that the MBSRP and conscious yoga reduced the systolic and diastolic blood pressures in our hypertensive patients in the post-test and follow-up compared to the control group. This finding is another confirmation of the effectiveness of the MBSRP in treating hypertension and psychological factors associated with it. The results of the present study are consistent with those of some previously conducted investigations.^19^^, ^^35^^, ^^36^ Hughes et al.^35^ showed that the MBSRP is effective in a group consisting of 56 men and women diagnosed with borderline hypertension not requiring medication. The patients receiving the MBSRP enhanced their physical and mental health, which prevented their blood pressure from increasing. The Blom et al.^36^ study on the effectiveness of the MBSRP with yoga and meditation in decreasing hypertension demonstrated significant differences in the post-test and at 3 months' follow-up. The authors suggested that mindfulness techniques be practiced continuously throughout the life and be turned into a style in life. In a study by Yuchy et al. ^37^ the effects of the MBSRP on a group of first-year nursing university students in variables such as the systolic blood pressure and anxiety symptoms were examined and the results indicated the effectiveness of this treatment on reducing the blood pressure and anxiety symptoms. This finding can be explained by the notion that group MBSRP and conscious yoga, through meditation techniques and mindfulness, can increase self-awareness and self-acceptance abilities, and thus improve the quality of life in patients. Mindfulness techniques (automatic exercise and focus) and relaxation training (body scan, relaxation and Hatha yoga) largely as a stress management skill must be practiced regularly and permanently in patients' lives.^25^ Studies have shown that mindfulness has been effective in improving mental and physical well-being and reducing physical symptoms.^14^ In explaining this finding it can be said that because mindfulness is a non-judgmental and balanced feeling of awareness that helps clarity and acceptance of emotions and physical phenomena,^34^ teaching its concepts to hypertensive patients who suffer from physical and mental problems will enable them to accept their feelings and physical symptoms better and as such reduce their level of sensitivity to the problem. In this regard, results show that emotional inhibition, as a negative emotion-regulating strategy, leads to cardiovascular diseases and hypertension, whereas cognitive evaluation of emotions, as a positive emotion-regulating strategy, leads to a reduction in the blood pressure.^15^ Another explanation for these results is that depression, anxiety, and stress are the main factors affecting patients with hypertension.^7^^, ^^9^ This finding can be explained by the notion that it causes a reduction in physical stress and produces epinephrine and norepinephrine hormones, thereby reducing stress and anxiety and, thus, the blood pressure in patients. Generally, mindfulness and emotion regulation are each reviewed, followed by a conceptual integration. Fundamental difficulties arising from the attempt to integrate the two domains are highlighted, especially as to the "reality" of thoughts, the relationship between thoughts and emotions, and the need to move beyond a valence model of emotion. Finally, a model is proposed outlining the likely critical processes and mechanisms that underlie "mindful emotion regulation.^38^ According to Park et al, mindfulness practices (relaxation, body scan, meditation, and yoga) lead to physiological changes and these changes are correlated with decreased activity of the sympathetic nervous system, they can reduce the blood pressure. The participants had a significantly lower respiratory rate during mindfulness meditation; however, in contrast to mindfulness meditation, controlled breathing alone did not reduce blood pressure, heart rate, or muscle sympathetic nerve activity. Mindfulness meditation acutely lowered blood pressure and heart rate in African-American males with hypertensive chronic kidney disease, and these hemodynamic effects might have been mediated by a reduction in sympathetic nerve activity. The respiratory rate was significantly lower during mindfulness meditation, but controlled breathing alone without concomitant meditation did not acutely alter hemodynamics or sympathetic activity in chronic kidney disease.^39^ Treatment in a group helps to enhance coping ability, sense of hope, and response to treatment; therefore, it can be argued that more therapeutic consequences are affected.^29^

In light of the results of the current study and similar ones, it can be concluded that the MBSRP and conscious yoga can be useful in reducing hypertension, promoting a healthy lifestyle, and encouraging the application of problem-focused coping strategies because of their emphasis on awareness and non-judgmental techniques. Nonetheless, it should be noted that the longevity of the effectiveness requires constant practice and expansion of mindfulness techniques throughout the life, such that this method can indeed become a lifestyle. 

The present study has some limitations, first and foremost amongst which are its relatively short follow-up period and its inability to perform a comprehensive sampling of all urban areas, limiting the scope to only one hospital. 

## Conclusion

The MBSRP can promote a healthy lifestyle, encourage problem-focused coping strategies, and decrease the systolic and diastolic blood pressures in patients with hypertension.

## Notes:

***This paper should be cited as:*** Nejati S, Zahiroddin A, Afrookhteh G, Rahmani S, Hoveida S. Effect of Group Mindfulness-Based Stress-Reduction Program and Conscious Yoga on Lifestyle, Coping Strategies, and Systolic and Diastolic Blood Pressures in Patients with Hypertension. J Teh Univ Heart Ctr 2015;10(3):140-148.
